# 

**Published:** 1967-12

**Authors:** 


### *Contents*

| | Page |
|---|---|
| THE COLLECTING OF WILD PLANTS with special reference to those of Medicinal or Toxicological Importance ... *T. F. Hewer* | 97 |
| A DISTRICT NURSE/GP ATTACHMENT ... *W. B. Fletcher* | 107 |

